# Heterogeneous nitrogen fixation rates confer energetic advantage and expanded ecological niche of unicellular diazotroph populations

**DOI:** 10.1038/s42003-020-0894-4

**Published:** 2020-04-14

**Authors:** Takako Masuda, Keisuke Inomura, Naoto Takahata, Takuhei Shiozaki, Yuji Sano, Curtis Deutsch, Ondřej Prášil, Ken Furuya

**Affiliations:** 1 Department of Aquatic Bioscience, The University of Tokyo, Yayoi, Bunkyo, Tokyo 113-8657 Japan; 2 Institute of Microbiology, The Czech Academy of Sciences, Opatovický mlýn, 379 01 Třeboň, Czech Republic; 3 School of Oceanography, University of Washington, Seattle, WA USA; 4 Atmosphere and Ocean Research Institute, The University of Tokyo, Kashiwanoha, Kashiwa-shi, Chiba, 277-8564 Japan; 5 Present Address: Global Change Research Institute, The Czech Academy of Sciences, 664 24 Drásov, Olomouc, Czech Republic; 6 Present Address: Graduate School of Science and Engineering, Soka University, Tangi, Hachioji, Tokyo 192-8577 Japan

**Keywords:** Microbiology, Microbial ecology, Cellular microbiology

## Abstract

Nitrogen fixing plankton provide nitrogen to fuel marine ecosystems and biogeochemical cycles but the factors that constrain their growth and habitat remain poorly understood. Here we investigate the importance of metabolic specialization in unicellular diazotroph populations, using laboratory experiments and model simulations. In clonal cultures of *Crocosphaera watsonii* and *Cyanothece* sp. spiked with ^15^N_2_, cellular ^15^N enrichment developed a bimodal distribution within colonies, indicating that N_2_ fixation was confined to a subpopulation. In a model of population metabolism, heterogeneous nitrogen (N_2_) fixation rates substantially reduce the respiration rate required to protect nitrogenase from O_2_. The energy savings from metabolic specialization is highest at slow growth rates, allowing populations to survive in deeper waters where light is low but nutrients are high. Our results suggest that heterogeneous N_2_ fixation in colonies of unicellular diazotrophs confers an energetic advantage that expands the ecological niche and may have facilitated the evolution of multicellular diazotrophs.

## Abstract (web summary)

Takako Masuda et al. show that individual cells in clonal populations of *Crocosphaera watsonii* and *Cyanothece sp* exhibit varied nitrogen fixation rates. This heterogeneity within the population decreases the energetic cost of respiration and expands the viable habitats for these unicellular diazotrophs.

## Introduction

Nitrogen (N_2_) fixing microorganisms (diazotrophs) are critical suppliers of bioavailable nitrogen (N) in the world’s oceans. The N_2_ fixed by these organisms supports cell growth, but also enters the food web through grazing by zooplankton and excretion of ammonium (${{{\mathrm{NH}}}}_4^ +$) or other dissolved nitrogenous compounds^1–5^. All diazotrophs have a N_2_ fixing enzyme complex, nitrogenase. Since most nitrogenase enzymes are irreversibly damaged by molecular oxygen^6,7^, diazotrophs separate photosynthesis and N_2_ fixation spatially or temporally^8,9^. Diazotrophs are taxonomically diverse and occupy distinct large-scale habitats^9–12^, suggesting there are multiple strategies for managing the energetic demands of photosynthesis, growth, and N_2_ fixation under a wide range of ocean conditions. However, the links between diverse physiological strategies and the resulting ecological niches and spatial distributions remain poorly understood^10,13–15^.

*Crocosphaera watsonii* (hereafter *Crocosphaera*), a marine unicellular diazotroph, is abundant and widespread in tropical and subtropical oceans^10,11,16,17^, and its areal N_2_ fixation rate (µmol N m^−2^ d^−1^) can be equal to or greater than that of *Trichodesmium*, a filamentous diazotroph abundant in tropical oceans^16,18,19^. Due to its strong diel alternation between C and N metabolisms, *Crocosphaera* is a promising model for investigating cellular C and N physiology^17,20–23^. *Cyanothece* is another well studied unicellular diazotroph. It is closely related to the sequence-defined genus, UCYN-C^24,25^, which has been observed to supply N to other phytoplankton and contribute to vertical POC (particulate organic carbon) transport^26^. Intensive studies on *Cyanothece* physiology make it a model organism to study the physiology of unicellular diazotrophic cyanobacteria^27,28^.

When exposed to a light:dark cycle, the peak of N_2_ fixation activity of most unicellular photosynthetic diazotrophs is restricted to the dark period. A similar diel cycle is observed in *Crocosphaera*^20–22,29^ and *Cyanothece*^27^, however, both taxa can be forced to fix N_2_, if maintained under constant illumination for an extended period^23,30,31^. This observation led to speculation that its metabolism is heterogeneously distributed among cells in a population^14^ as observed in *Trichodesmium*^8^.

Recent technological advances in the visualization of enriched stable isotopes in individual cells using NanoSIMS enable cell level analyses of N_2_ fixation activity^32–38^. With this technology, high variations in C and N_2_ fixation activity from in situ “*Crocosphaera*-like” cell colonies were shown, suggesting heterogeneity of metabolisms^17^. During the same period of time, similar physiological heterogeneity was observed among the clonal population of *Crocosphaera* (WH8501)^23^. These observations lead us to question how widely this heterogeneity applies and how it impacts the cellular energetics and resulting ecological niches.

Here, we investigated physiological heterogeneity among clonal populations of multiple genera of cyanobacteria, *Crocosphaera* (PS0609A) and *Cyanothece* (ATCC51142), by quantifying the pattern in N_2_ fixation and C uptake at the sub-cellular level. Using clonal populations prevents interference from other N fixing organisms, a potential problem noted in the in situ study^17^ and using different genera and strains of cyanobacteria allows us to evaluate the generality of the pattern. We have also applied multiple statistical methods to quantify the heterogeneity in C and N_2_ fixation. To analyze the energetics and C consumption of the observed cell-level heterogeneity, we present a model of diazotroph population to simulate the advantage of maintaining both nitrogen fixing and non-N_2_ fixing cells. We used oceanographic data to predict the implications of metabolically differentiated populations for the ecological niche of unicellular diazotrophs through the photic zone.

## Results

### Heterogeneity in N enrichment among cells

**Fig. 1:**
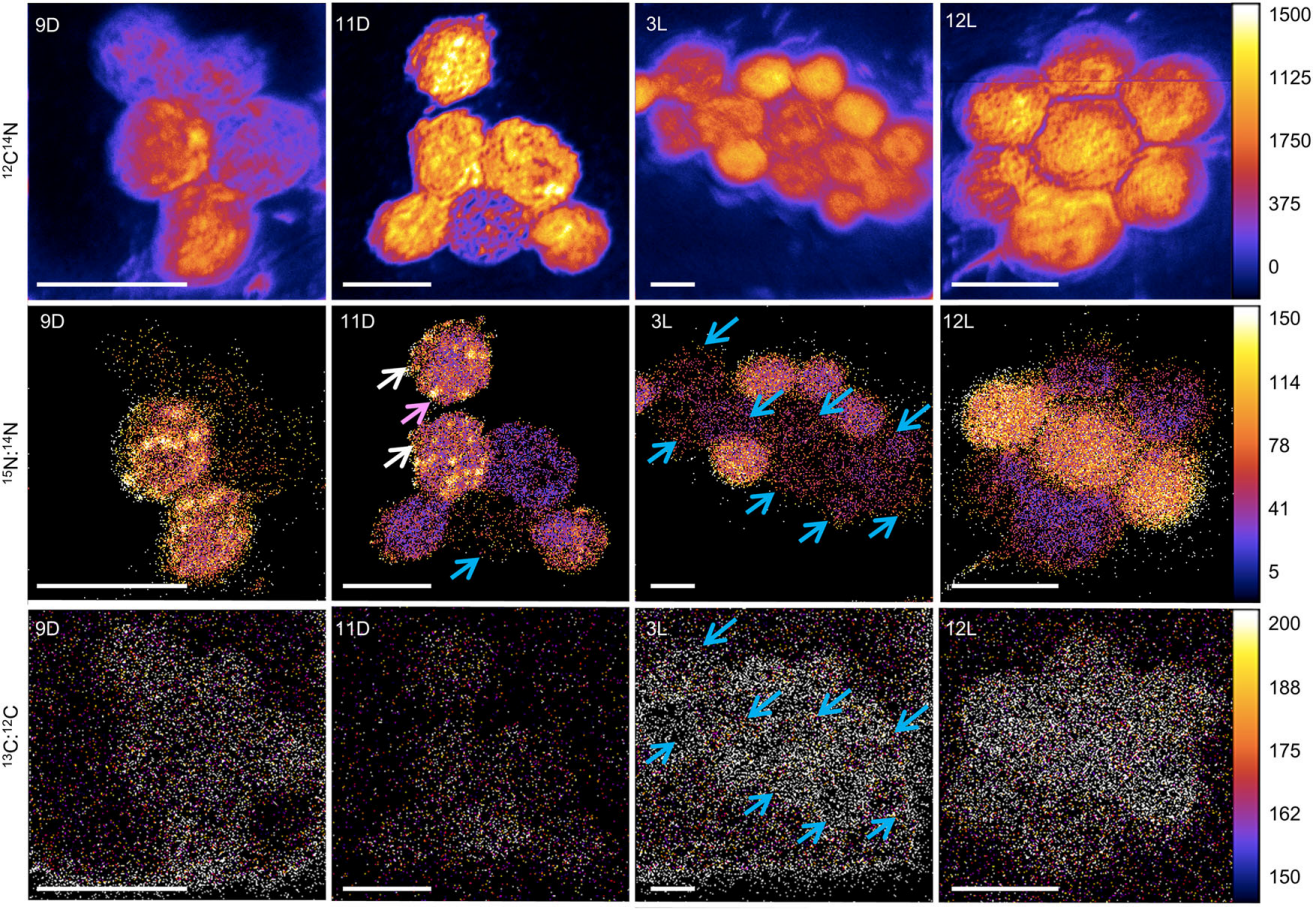
NanoSIMS images showing temporal changes in isotopic enrichment in *Crocosphaera* under a dark:light cycle of 12:12 h. Numerals and alphabets in each photo denote time in a photoperiod: 9D and 3 L indicate 9 h in the dark and 3 h in the light, respectively. The colored bars for ^12^C^14^N indicate the number of ions collected per pixel, the other colored bars indicate the ratio of ^15^N: ^14^N and ^13^C:^12^C with the scale factor of ×10,000. White arrows in ^15^N:^14^N at 11D show the cells with intensive ^15^N enrichment, blue arrow shows the cells with less ^15^N enrichment, pink arrows show ^15^N hotspots. Scale bars, 5 µm. ^12^C^14^N shows the baseline, from which labeling departs in lower panels; ^15^N:^14^N shows the fate of newly fixed ^15^N_2_, ^13^C:^12^C shows newly fixed ^13^C uptake.

**Fig. 2:**
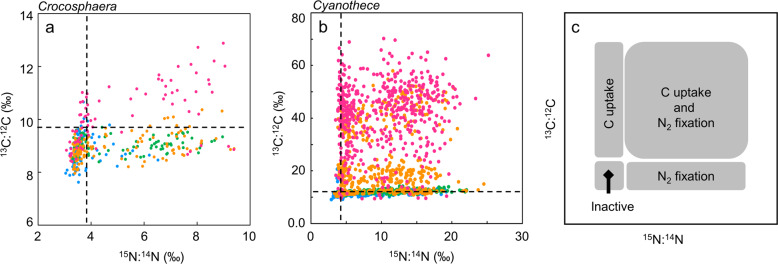
^13^C and ^15^N uptake by two different unicellular diazotrophs. Nitrogen and carbon incorporation by **a** *Crocosphaera* and **b** *Cyanothece* shown by scatter plot of ^15^N:^14^N and ^13^C:^12^C in individual cells. Black dot-lines show threshold of ^15^N and ^13^C enrichment. **c** Schematic view of scatter plot. Blue, data obtained during 0D6D; Green, data obtained during 7D-12D; Orange; data obtained 1L-6L; Pink, data obtained 7L-12L. Higher ^13^C uptake in *Crocospharea* compared to *Cyanothece* is likely to reflect higher initial ^13^C enrichment in the culture for *Crocosphaera*: 9.7 atm% for *Crocosphaera* and 1.7 atm% for *Cyanothece* (see Methods).

**Fig. 3:**
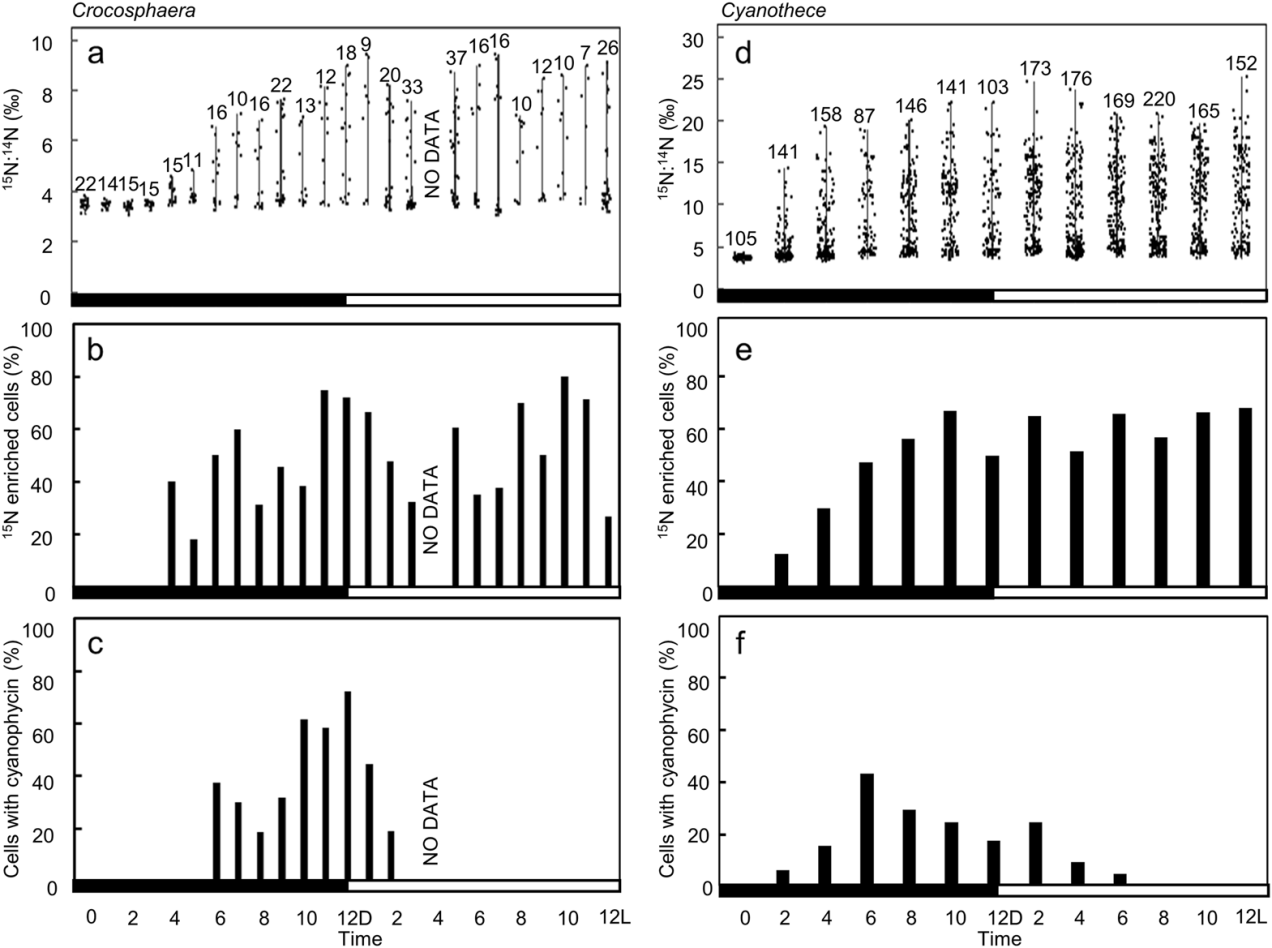
Observed heterogeneity in ^15^N uptake by two different unicellular diazotrophs. **a**, **d** Diel change in N_2_ fixation (^15^N:^14^N), **b**, **e** diel change in percentage of ^15^N-enriched cells, **c**, **f** diel change in percentage of cells with ^15^N hotspot in **a** to **c** *Crocosphaera* and **d** to **f** *Cyanothece*. Black and white bars at the bottom of each graph indicate dark and light period, respectively. NO DATA at 4 L in *Crocosphaera* shows no data was collected.

^15^N enrichment was variable within a single strain of continuous culture grown *Crocosphaera* cells (Figs. 1 and 2) and batch culture grown *Cyanothece* (Fig. 2). Initially, ratios of ^13^C:^12^C (=^13^C/^12^C) and ^15^N:^14^N (=^15^N/^14^N) were 8.8 ± 0.5‰ and 3.5 ± 0.1‰, respectively in *Crocosphaera* harvested at steady state under continuous culture and were 11.0 ± 0.4‰ and 3.6 ± 0.2‰, respectively in *Cyanothece* harvested at exponential phase under batch culture (Fig. 3 and Supplementary Table 1). After 11 h in the dark, two cells (Fig. 1; white arrows in panel 11D ^15^N:^14^N) were more strongly enriched in ^15^N compared to the other four cells, of which one cell showed the least enrichment (Fig. 1: blue arrow). However, the least ^15^N enriched cells were actively ^13^C enriched in the light period (e.g., cells with blue arrow in panel 3L ^13^C:^12^C) showing that these cells were alive and metabolically active. The variable ^15^N enrichment was observed not only under continuous culture but also under exponentially growing batch culture and suggests that heterogeneous ^15^N_2_ fixation happens with or without nutrient stress.

The ^15^N_2_ fixation in the dark was observed based on the temporal changes in cellular ^15^N:^14^N ratios in both *Crocosphaera* and *Cyanothece* (Fig. 3a, d and Supplementary Table 1). Calculated per-cell ^15^N enrichment rates, *ρ*, followed similar trends as the ^15^N:^14^N ratio, and increased significantly (*p* = 0.014 by one-way ANOVA^39^) in *Crocosphaera* from 0 fmol N cell^−1^ h^−1^ at the start of the dark period to a mean of 17.3 fmol N cell^−1^ h^−1^ at 7D, but with a range of 0 fmol N cell^−1^ h^−1^ to 37.7 fmol N cell^−1^ h^−1^ (Supplementary Table 1b). These values are comparable to those reported in earlier studies (Supplementary Table 2). *Cyanothece* also showed a similar trend within population heterogeneity; ρ varied from 0 fmol N cell^−1^ h^−1^ to 12.7 fmol N cell^−1^ h^−1^ across 84 cells at the time of highest mean ^15^N enrichment (5.54 fmol N cell^−1^ h^−1^ at 6D) (Supplementary Table 1b). The ^15^N:^14^N ratios measured by NanoSIMS were in good agreement with the ratios measured by mass spectrometer (Supplementary Fig. 1).

The proportion of *Crocosphaera* cells that incorporated detectable ^15^N (i.e., cells with ^15^N:^14^N exceeding 2 SD above the mean at time 0: 3.8‰ for *Crocosphaera*, 4.0‰ for *Cyanothece*) increased from 40 to 75% in the dark, suggesting that at least ~25% of cells did not detectably fix N_2_ (Fig. 3b, e). Higher variability of ^15^N enriched cells in *Crocosphaera* compared to *Cyanothece* may be the result of low number of observed cells (between 7 to 33 cells at each time point) (Fig. 3b, d). Hotspots of ^15^N:^14^N were observed in the dark period. The ^15^N hotspots started to appear after 5D, and continued to form until the beginning of the light period (2 L), with the peak of 62% at 10D (Fig. 1 and 3c) in *Crocosphaera*. Similar temporal changes were observed for the proportion of cells with ^15^N hotspot among total cells in *Cyanothece*, from 2D to 6 L with a peak at 6D. Therefore, the lack of ^15^N hotspot in at least ~40% of cells again shows that a large fraction of cells did not detectably fix N_2_. N_2_ fixation earlier in the diel cycle in *Cyanothece* compared to *Crocosphaera* (Fig. 3) supports previous reports of a peak around 4D in *Cyanothece* and 9D in *Crocosphaera* under 12 L:12D cycle^21,40^.

To quantify the differentiation of rates within each population, we examined the statistical distribution of C and N isotope enrichments among all cells. Intercellular metabolic heterogeneity was defined as the coefficient of variation (CV; ref. ^41^) in each isotope ratio. The variations in ^15^N enrichment are observable in cell level ^15^N:^14^N ratios, which varied from 3.2‰ to 6.2‰ (4.4 ± 1.0‰, CV = 23.6%) at 6D in *Crocosphaera*, 3.5‰ to 18.9‰ (8.3‰ ± 4.4‰, CV = 53.8%) at 6D in *Cyanothece* (Figs. 2 and 3 and Supplementary Table 1). In contrast, ^13^C uptake (^13^C:^12^C ratio) was generally similar across cells, in both ^15^N-enriched cells and non-enriched cells (Figs. 1 and 2 and Supplementary Table 1). For example, in the ^13^C:^12^C ratio of 3 L in Fig. 1, all 14 cells are enriched similarly, with ^13^C:^12^C ratios between 7.9‰ and 9.4‰ (8.7‰ ± 0.5‰, CV = 6.2%) in *Crocosphaera* (Fig. 2). The CV for ^15^N:^14^N (23.6 to 31.4% during 6D to 12D, 25.4 to 48.0% during 6 L to 12 L) were greater than those estimated for ^13^C:^12^C during 6 L to 12 L (4.8 to 10.6%), suggesting higher heterogeneity in ^15^N_2_ fixation compared to ^13^C fixation. The same trend was observed in *Cyanothece* (46.4 to 56.2% during 6D to 12D, 45.5 to 48.9% during 6 L to 12 L in ^15^N:^14^N, 24.0 to 40.1% in ^13^C:^12^C during 6 L to 12 L) (Supplementary Table 1a).

**Fig. 4:**
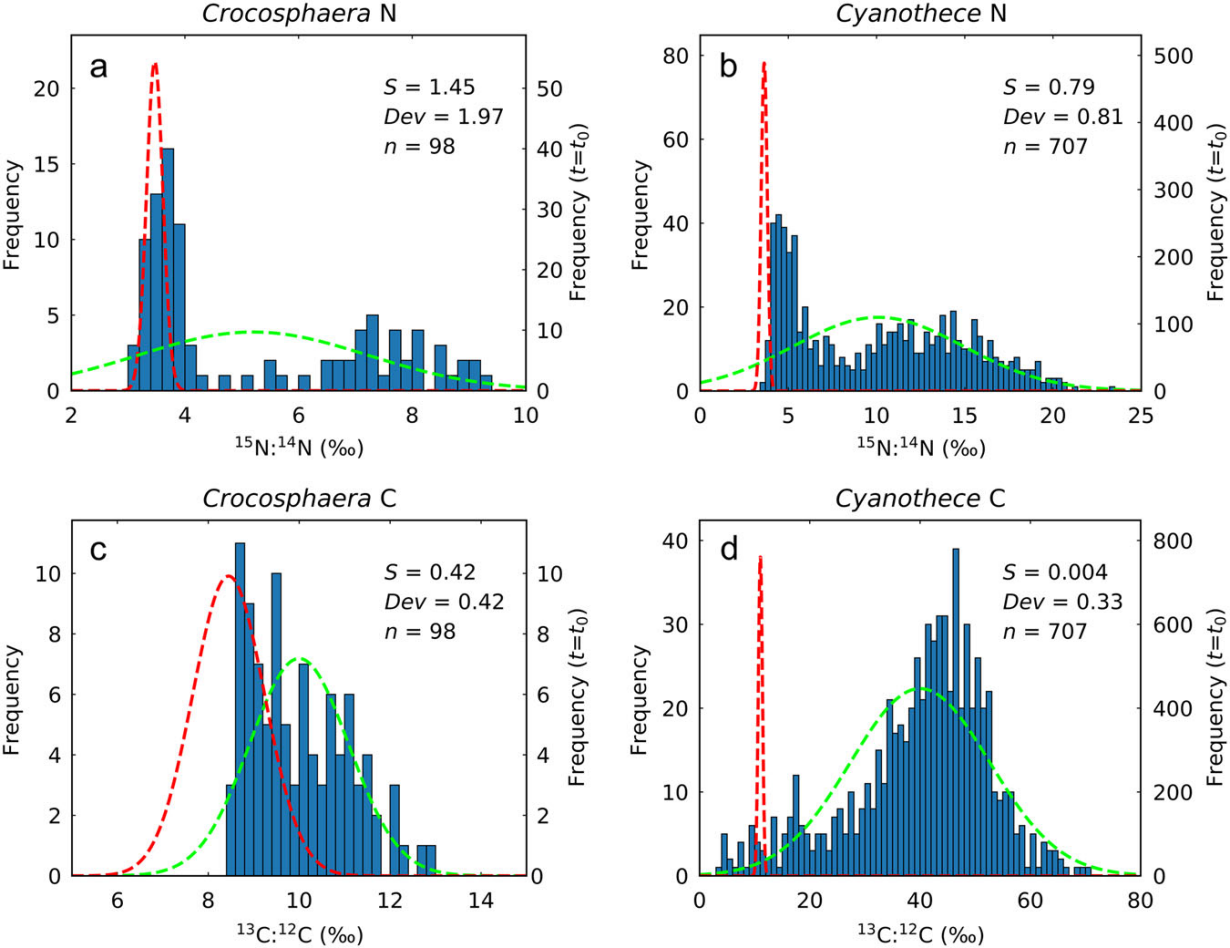
Statistical analysis of heterogeneous uptake of N and C. Frequency distribution of **a**, **b** ^15^N:^14^N for *Crocosphaera* and *Cyanothece* (6 L to 12 L) and **c**, **d** ^13^C:^12^C for *Crocosphaera* and *Cyanothece* (6 L to 12 L), respectively. Red dashed curve is the normal distribution for the initial condition (*t* = *t*_0_) with the special *y*-axis on the right (that on the left is for other plotted values). Green dashed curve is the normal distribution based on the mean value and standard deviation of the probability density. *S*, bimodal separation; *Dev*, deviation from the normal distribution; *n*, number of samples. Both for *Crocosphaera* and *Cyanothece*, *S* and *Dev* are larger for ^15^N:^14^N, suggesting stronger heterogeneity for N uptake.

The distribution of isotope ratios among cells reveals qualitatively different enrichment trends for C compared to N, for both *Crocosphaera* and *Cyanothece* (Fig. 4). The distribution of ^15^N:^14^N reveals two distinct peaks after 12 h, one that remains near the initial ratio and a second that develops at enriched levels of ^15^N, for both *Crocosphaera* and *Cyanothece* (Fig. 4a, b). In contrast, few cells remain at the initial ratio ^13^C:^12^C (Fig. 4c, d), and only a single broad peak is evident. To evaluate this bimodality, we calculated the “bimodal separation” (*S*; ref. ^42^), a distance between the means of two Gaussian distributions fit to the data (see Methods). The separations of peaks in ^15^N:^14^N was consistently larger than for ^13^C:^12^C, both for *Crocosphaera* (*S* = 1.45 for N, vs. *S* = 0.42 for C) and for *Cyanothece* (*S* = 0.79 for N, vs. *S* = 0.004 for C). We have also applied the bimodal curve fitted with ^15^N:^14^N to ^13^C:^12^C with the curve shape maintained (relative relation between two normal distributions and *S* are maintained); even after the curve is fitted to ^13^C:^12^C, the difference between the data and the curve is statistically significant for both diazotrophs (*p* < 0.001), indicating a significant difference between ^15^N:^14^N and ^13^C:^12^C.

To confirm that the distribution of N isotopes develops a bimodal structure indicative of distinct rates among sub-populations, we compared the observed frequency distributions to a single Gaussian distribution with the same mean value and standard deviation (Fig. 4). For ^15^N:^14^N, the peak of the normal distribution appears near the local minima between the two peaks of the data (Fig. 4a, b), again indicating strong bimodal separation. In contrast, the normal distribution largely overlaps with the data for ^13^C:^12^C (Fig. 4c, d). We computed the deviation (*Dev*) from the normal distribution by adapting a commonly used form of Chi square (*χ*^2^) normalized by the sample number (*n*) (see Methods). The deviations from a single Gaussian distribution are stronger for ^15^N:^14^N than for ^13^C:^12^C for both diazotrophs (*Dev* = 1.97 vs. 0.42 for *Crocosphaera* and 0.81 vs. 0.33 for *Cyanothece*). These results qualitatively and quantitatively support stronger heterogeneity in N uptake than for C uptake, indicating a clear separation between N_2_ fixing cells and non-N_2_ fixing cells, without a comparable separation of C fixation.

Although *Crocosphaera* and *Cyanothece* are generally referred to as free-living unicellular cyanobacteria, they have been reported in colonies of more than two cells^17,23^ (Fig. 1). In the *Crocosphaera* culture, 55% of total cells were observed as colonies of 3 to 5 cells in this measurement (Supplementary Fig. 2). Colonial *Crocosphaera* cells were shown in earlier culture studies^23^, and an in situ study found that 45 to 85% of *Crocosphaera*-like cells were observed as colonies of 3 to 242 cells^17^. Colonial formation of cells might increase the efficiency of excreted ${{{\mathrm{NH}}}}_4^ +$ transfer among cells.

### Simulating population heterogeneity of N_2_ fixation

The strong concentration of newly fixed N in a sub-population of colonial unicellular diazotrophs suggests that localizing the costly process of N_2_ fixation may confer an advantage to the population as a whole. A large part of the energetic cost of N_2_ fixation is incurred in the protection of nitrogenase from O_2_, which is achieved through excess respiration of C (refs. ^43,44^). We therefore hypothesize that having only a limited proportion of cells to pay the oxygen management cost could reduce community C requirements, potentially leading to overall higher growth.

**Fig. 5:**
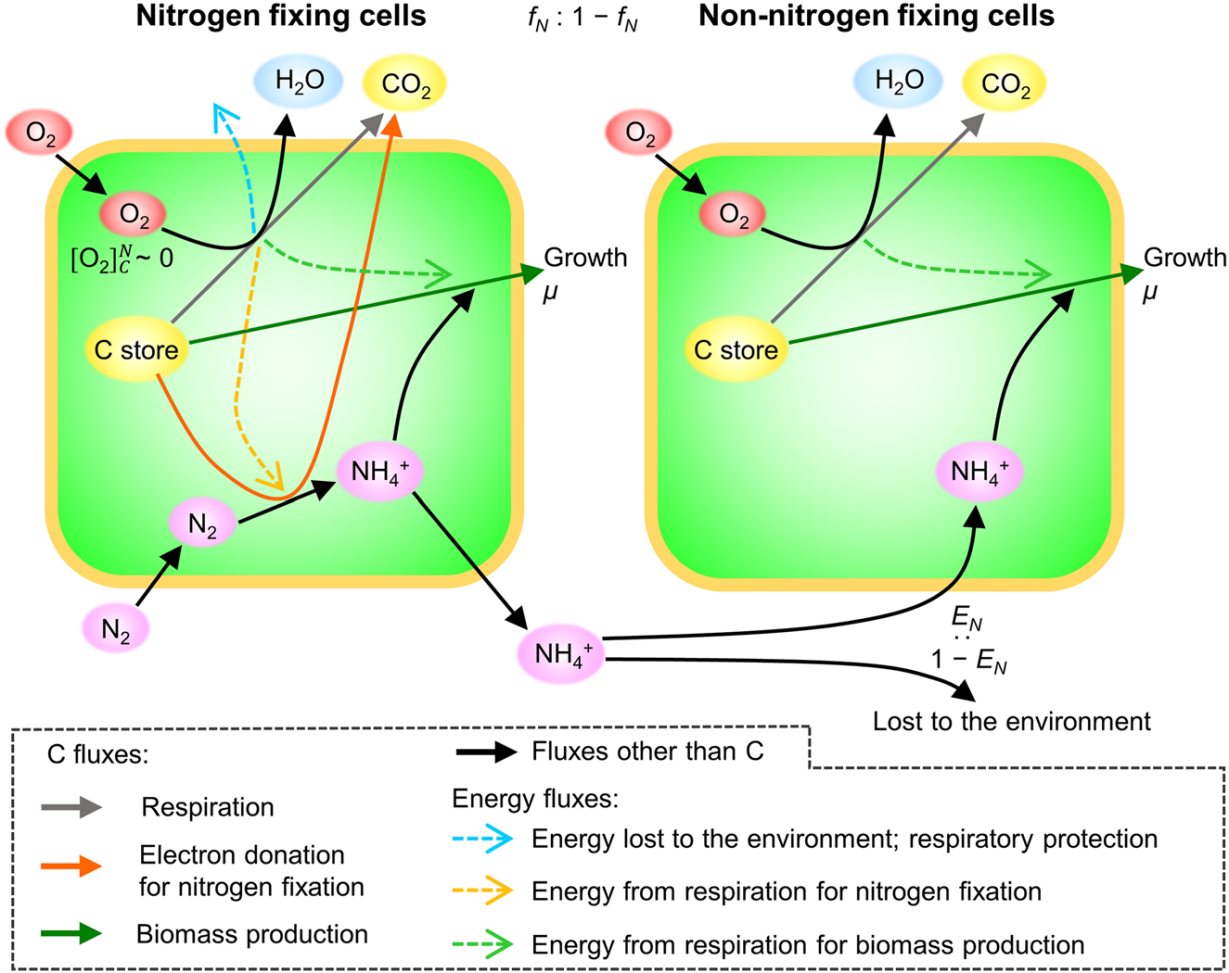
Schematic of cell flux model simulating heterogeneity of *Crocosphaera* during the dark period. Green space, cytoplasmic space; peach frames, cell membrane layers; circular blobs, chemical compounds; solid arrows, material fluxes; dashed arrows, energy fluxes. C store represents C storage accumulated during the preceding light period, which is used for multiple purposes. The use of C store is represented by solid arrows of different colors by C fluxes and the different energy fluxes from respiration are colored differently; see the list at the bottom. *f*_*N*_ represents the fraction of N_2_ fixing cells; thus that of non-nitrogen-fixing cells becomes 1 − *f*_*N*_. The O_2_ concentration of N_2_ fixing cell $[{{{\mathrm{O}}}}_2^{}]_C^N$ is kept small through respiratory protection at the expense of C store. Contrarily, only biosynthetic respiration occurs in non- N_2_ fixing cells. Excreted^,^ fixed N$\left( {{{{\mathrm{NH}}}}_4^ + } \right)$ is transferred to non-N_2_-fixing cells with efficiency of *E*_*N*_; 1 − *E*_*N*_ is the fraction of excreted NH_4_^+^ lost to the environment. Cells grow at the rate of *μ* (d^−1^).

To evaluate the potential benefits of confining N_2_ fixation to a sub-population, we used a Cell Flux Model of a N_2_ fixer^44^. The model uses a coarse-grained metabolic flux network including core metabolisms of respiration, biosynthesis and N_2_ fixation, which are constrained by mass, electron and energy balance (Fig. 5) (see Methods for details). We simulate a steady state environment where cells grow at a rate of *μ* (d^−1^). To maintain the prescribed rate of growth, energy must be provided by respiration, with distinct rates allocated to N_2_ fixation and biomass production^44,45^. In turn, the total respiration rate predicts the intracellular O_2_ concentrations, for a given diffusivity of O_2_ across the cell membrane. Additional respiration is added as needed to maintain anoxia inside the cell, thus protecting the nitrogenase enzyme and enabling N_2_ fixation^44^. The total carbon consumption rate per cell is computed to satisfy the sum of all 3 demands: biomass growth, N_2_ fixation, and respiratory protection against O_2_.

Here we adapted this cellular model^44^ to represent a heterogeneous colony of cells (the model version named CFM-Colony, with a fraction *f*_*N*_ that fix N_2_, and a remaining fraction 1 − *f*_*N*_, that do not. The two sub-populations share a common medium, allowing N_2_-fixing cells to transfer fixed nitrogen $\left( {{{{\mathrm{NH}}}}_4^ + } \right)$ to non- N_2_-fixing cells. The transfer of newly fixed N is prescribed by an efficiency parameter, *E*_*N*_, with the remaining fraction (1 − *E*_*N*_) of excreted ${{{\mathrm{NH}}}}_4^ +$ being lost from the entire colony.

To quantify the impact of heterogeneous rates of N_2_ fixation, we compare its population-scale rate of C consumption (denoted *C*_*S*_), to the rate that would apply to a homogeneous population of the same size (denoted $C_S^0$). When $C_S/C_S^0 \, < \, 1$, the colony has lower C consumption with heterogeneous N_2_ fixation than homogeneous N_2_ fixation. The rate of N_2_ fixation by a heterogeneous community, *N*_*S*_, relative to a population with uniform rates, $N_S^0$ (when *f*_*N*_ = 1) can be expressed as follows:

1 $$N_S/N_S^0 = f_N\left( {1 + \frac{{1 - f_N}}{{f_NE_N}}} \right)$$

The ratio of C consumption associated with N_2_ fixation and respiratory protection follows the ratio of N fixation rates by heterogeneous versus homogeneous populations (Eq. 1).

**Fig. 6:**
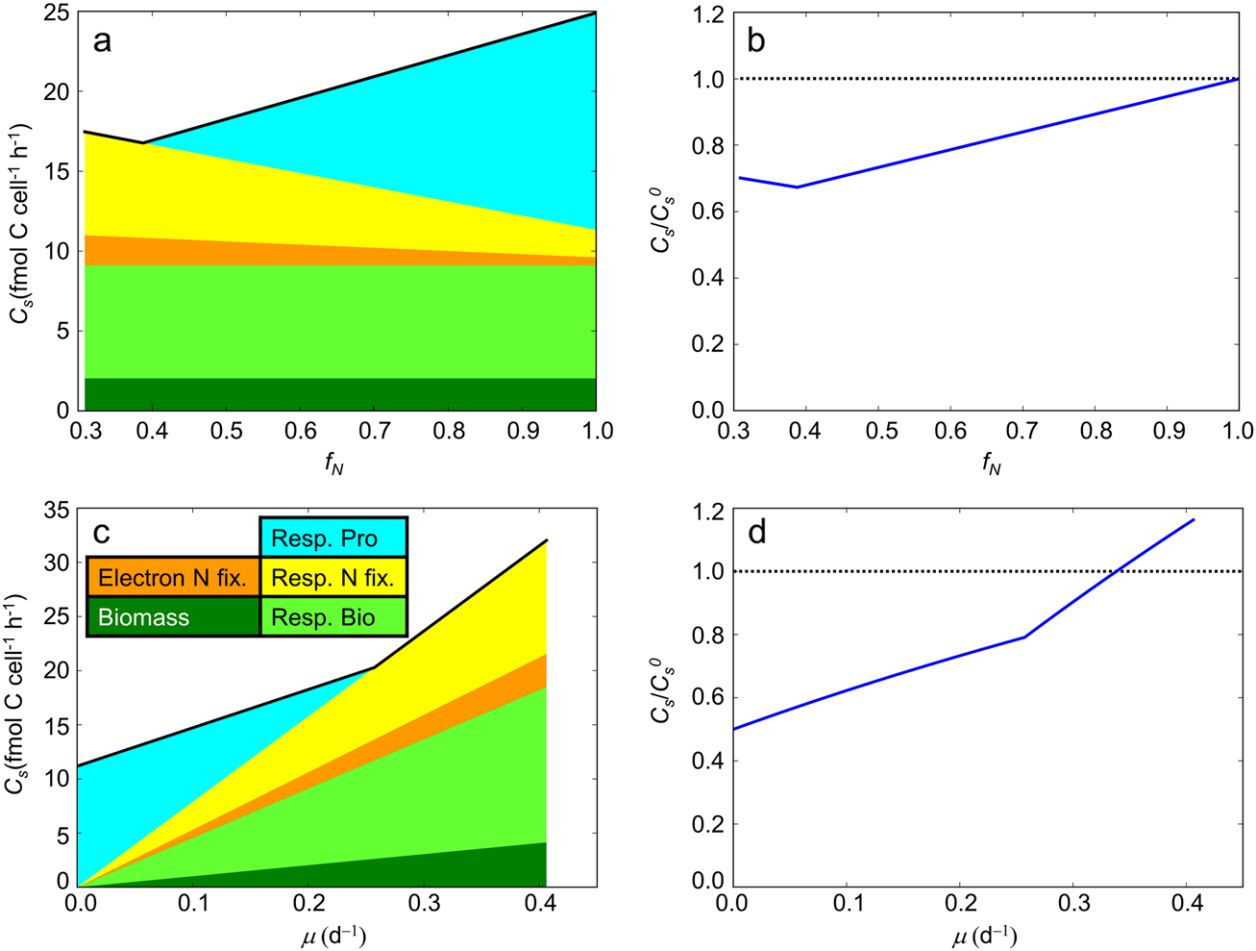
Carbon use of heterogeneous population, *C*_*S*_ and *C*_*S*_ relative to non-heterogeneous population ${{{\boldsymbol{C}}}}_{{{\boldsymbol{S}}}}^{{{\boldsymbol{0}}}}$. **a** *C*_*S*_ for various *f*_*N*_. **b** $C_S/C_S^0$ for various *f*_*N*_. **c** *C*_*S*_ for various *μ*. **d** $C_S/C_S^0$ for various *μ*. For **a** and **c** the legend in **c** shows the colors used for each fluxes; Dark green, biosynthesis; Bright green, respiratory energy production for biosynthesis; Orange, electron donation for N_2_ fixation; Yellow, respiratory energy production for N_2_ fixation; Cyan, respiratory protection. See Fig. 5 for more detail where similar colors are used for each C flux. Black solid lines at the top of **a** and **c** represent the total C fluxes. Black dotted lines in **b** and **d** are for $C_S/C_S^0 = 1$. *f*_*N*_ = 0.5, *E*_*N*_ = 0.2, and *μ* = 0.2 (d^−1^) unless they are variable on the *x*-axes. Temperature *T* = 26 °C and O_2_ concentration in the environment [O_2_] = 208 µM, representing saturated concentration at this temperature and salinity of 35ppt^81^.

Modeled colonies with N_2_ fixation confined to a sub-population benefit from a substantial drop in overall C consumption, due to lower community level requirements for respiratory protection of nitrogenase (Fig. 6). For typical *Crocosphaera* growth rates (*μ* = 0.2) and a low efficiency of ${{{\mathrm{NH}}}}_4^ +$ transfer (*E*_*N*_ = 0.2) C savings amount to ~8 fmol C cell^−1^ h^−1^, which is >30% of the C budget of a population with homogeneous rates (Fig. 6a, b). Total C consumption reaches a minimum value at an intermediate value of *f*_*N*_, due to two opposing factors; as *f*_*N*_ initially decreases below 1, respiratory protection is reduced. However, as *f*_*N*_ decreases, a larger portion of cells must also rely on transferred ${{{\mathrm{NH}}}}_4^ +$, which allows more ${{{\mathrm{NH}}}}_4^ +$ to be dissipated into the environment, requiring higher C consumption to replace it. This effect is represented by (Eq. 1) where increasing *f*_*N*_ leads to increasing *N*_*S*_. At an intermediate value of *f*_*N*_, these two factors minimize *Cs*, and respiratory protection is covered by energetically balanced productive flows of respiration.

**Fig. 7:**
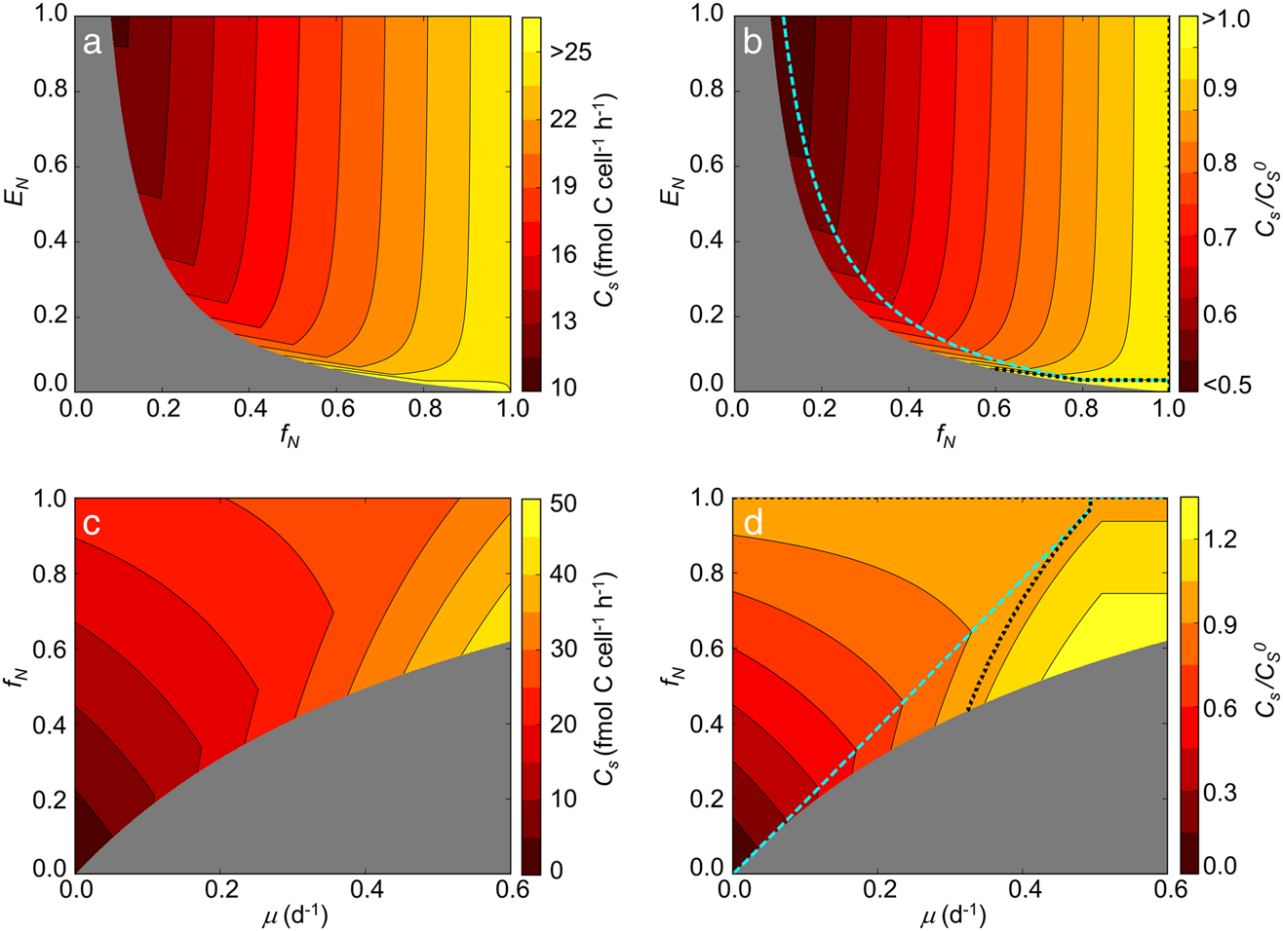
Carbon use of heterogeneous population, *C*_*S*_ and *C*_*S*_ relative to non-heterogeneous population ${{{\boldsymbol{C}}}}_{{{\boldsymbol{S}}}}^{{{\boldsymbol{0}}}}$ plotted for multiple parameters. **a** *C*_*S*_ for various *f*_*N*_ and *E*_*N*_. **b** $C_S/C_S^0$ for various *f*_*N*_ and *E*_*N*_. **c** *C*_*S*_ for various *μ* and *f*_*N*_. **d** $C_S/C_S^0$ for various *μ* and *f*_*N*_. In **b** and **d**, dashed lines in cyan indicates optimum *f*_*N*_, which gives lowest $C_S/C_S^0$ for *E*_*N*_ and *μ*, respectively. Dotted lines indicate where $C_S/C_S^0 = 1$ (note that $C_S/C_S^0$ is always 1 at *f*_*N*_ = 1). Gray zones indicates where N_2_ fixing capacity cannot sustain the population. *f*_*N*_ = 0.5, *E*_*N*_ = 0.2, and *μ* = 0.2 (d^−1^) unless they are variable on the axes. Temperature *T* = 26 °C and O_2_ concentration in the environment [O_2_] = 208 µM, representing saturated concentration at this temperature and salinity of 35 ppt (ref. ^81^).

The value of *f*_*N*_ that maximizes C savings tends to increase with decreasing *E*_*N*_ due to increased costs for N_2_ fixation [Eq. 1] (Fig. 7b). When *E*_*N*_ = 0.1, *C*_*S*_ (thus $C_S/C_S^0$) reaches a minimum at *f*_*N*_ ~ 0.56 (Supplementary Fig. 3), a level of heterogeneity similar to that seen in the culture experiments, in which about a half of cells fix N_2_. This optimum *f*_*N*_ also increases with the growth rate *µ* due to increased energy costs for biomass production and N_2_ fixation (Fig. 7d). The 2D plot of *C*_*S*_ and $C_S/C_S^0$ for various *f*_*N*_ and *E*_*N*_ shows that up to 55% of C can be saved at high *E*_*N*_ and low *f*_*N*_ (Fig. 7a, b). On the other hand, even at *E*_*N*_ < 0.1, heterogeneity can still save carbon (Fig. 7b), due to the small cost of N_2_ fixation relative to respiratory protection^44^. Considering the fact that C is one of the limiting factors for the growth for diazotrophs^28,46,47^, heterogeneity of N_2_ fixation might be an important strategy to increase their growth rates.

Because unicellular diazotrophs can use ${{{\mathrm{NH}}}}_4^ +$, growth efficiency should be maximized when cells can meet their N demand from ${{{\mathrm{NH}}}}_4^ +$ in the environment, thus saving the considerable cost of N_2_ fixation (Fig. 6a, c). If cells rely solely on the N_2_ fixation for their N source, higher growth rate would render respiratory protection negligible, yielding higher growth efficiency. For example, the cell flux model (Fig. 6) predicts that as growth rate increases beyond ~0.28 (d^−1^), respiratory protection is no longer needed and the growth efficiency reaches its highest level. This occurs at a specific *f*_*N*_ where respiratory protection is minimized with minimum loss of N to the environment (*C*_*S*_ and $C_S/C_S^0$ at *f*_*N*_ ~ 0.38 in Fig. 6a, b respectively and cyan dashed curve in Fig. 7b).

The amount of C saved by heterogeneous N_2_ fixation depends only slightly on the poorly known value of *E*_*N*_. This insensitivity is based on the relatively small cost for N_2_ fixation^44^. While N_2_ fixation requires 16 ATP per N_2_, when *E*_*N*_ = 1, the cost is predicted to be low relative to the whole cell energy requirement for biosynthesis since N_2_ fixation is just one reaction and there are many other pathways where ATP is consumed in the process of biosynthesis. In addition, cost for O_2_ management is overwhelming. As *E*_*N*_ decreases, the cost for N_2_ fixation increases inversely proportional to *E*_*N*_, but due to the relatively low costs of N_2_ fixation, the whole cell C costs (thus *C*_*S*_ and $C_S/C_S^0$) are relatively insensitive to *E*_*N*_.

The energetic advantage of heterogeneous N_2_ fixation rates increases as growth rates decline (Figs. 6c, d and 7c, d). Slower growth rates reduce the costs of biomass synthesis and N_2_ fixation, thus making respiratory protection a dominant energetic and C cost (Fig. 6c). Since heterogeneous populations can lower this cost by focusing N_2_ fixation in a fraction of cells, more C can be saved at lower *μ*. Over 90% of C can be saved at low *μ* and low *f*_*N*_ (Fig. 7d). On the other hand, when *μ* > 0.35 (d^−1^), $C_S/C_S^0$ can go above 1 (Figs. 6d and 7d) due to high costs for growth and N_2_ fixation, and N loss to the environment. The growth rates of *Crocosphaera* compiled from laboratory studies have a mean value of *μ* < 0.3 (d^−1^) (ref. ^48^). In the ocean, nutrients such as iron and phosphorus are generally more limited compared to culture conditions leading to even lower *μ*. Thus, with a typical growth rate in the ocean, it is likely that population heterogeneity in N_2_ fixation can save a considerable fraction of population C costs.

### Implications for vertical habitat range

Fixed C is required for N_2_ fixation, respiration and cellular growth, providing energy, electrons and reduced C. In the open subtropical ocean, chlorophyll concentrations typically reach a maximum at the bottom of the photic zone, and the top of the nutricline, ~100 m depth, where both light and nutrients are adequate for growth, albeit at low rates. Below these depths, available light becomes so low that it prevents cells from fixing enough C to be viable (here we define maximum viable depth, MVD). Since heterogeneous N_2_ fixation reduces the overall C requirement of such populations, it could act to extend their MVD deeper into the nutricline.

**Fig. 8:**
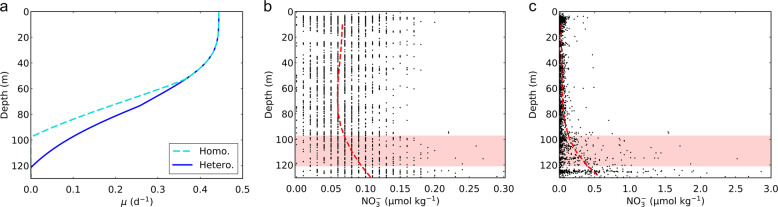
Viability range of *Crocosphaera* in the water column expanded in depth by heterogeneous N_2_ fixation. **a** Light dependent growth rate (*µ*) of populations of homogeneous (cyan dashed curve: Homo.) and heterogeneous N_2_ fixation (blue solid curve: Hetero.). **b**, **c** observed ${{{\mathrm{PO}}}}_4^{3 - }$ and ${{{\mathrm{NO}}}}_3^ -$ concentrations, respectively, from the Hawaii Ocean Time-series (HOT); 22° 45’N, 158° 00’W (ref. ^49^). Data are based on 25 years of observations (from 1988 to 2012); red dashed curves represent the averaged values. The red shading represents the difference of the depth where *µ* becomes zero between the two different populations in **a**; heterogeneous N_2_ fixation allows *Crocosphaera* to utilize higher concentration of ${{{\mathrm{PO}}}}_4^{3 - }$.

We simulated the depth variation of the growth rate for *Crocosphaera* populations with homogeneous versus heterogeneous N_2_ fixation rates (see Methods). The model result shows that MVD of the heterogeneous population is ~ 25 m deeper than that of the homogeneous population (Fig. 8a). This expanded MVD may be important because the available nutrient typically increase with depth and expanding MVD allows *Crocosphaera* to utilize the higher concentration of the growth-essential nutrient. For example, at the Hawaii Ocean Time-series (HOT) site at 22° 45’N, 158° 00’W (ref. ^49^), the concentration of phosphate (${{{\mathrm{PO}}}}_4^{3 - }$: one of the potentially limiting nutrients) increases below ~80 m depth and heterogeneous populations would be able to utilize ~40% higher concentration than homogeneous populations (Fig. 8b). A similar depth profile of ${{{\mathrm{PO}}}}_4^{3 - }$ is observed in the South Pacific Gyre at 25°S, 170°W, where the highest *nifH* gene concentration of *Crocosphaera* have also been observed^50^. Under those conditions, the model predicts heterogeneous population would utilize up to ~90% higher concentration of ${{{\mathrm{PO}}}}_4^{3 - }$ (Supplementary Fig. 4a).

In these observations, ${{{\mathrm{NO}}}}_3^ -$ concentrations also increase with depth, which may lead to a partial suppression of N_2_ fixation (Fig. 8c and Supplementary Fig. 4b). However, the ${{{\mathrm{NO}}}}_3^ -$ concentrations in the expanded vertical niche remain well below what would cause full suppression^51–53^. For example, the concentration of ${{{\mathrm{NO}}}}_3^ -$ at MVD is below 2 µM whereas even 5 µM does not fully suppress N_2_ fixation of *Crocosphaera*^51–53^. Since respiratory protection is required regardless of the level of N_2_ fixation (thus required even when N_2_ fixation is partially suppressed)^54^, heterogeneous population would still save C even at depths near the MVD.

## Discussion

The results of our laboratory observations demonstrate that unicellular diazotrophic cyanobacteria form colonies in which the key metabolic function of N_2_ fixation is confined to a distinct subpopulation. Guided by these observations, metabolic modeling shows that this functional specialization may provide an energetic advantage, especially in oligotrophic regions where nutrient availability increases as light diminishes. These findings have important implications for role of metabolic specialization in the evolution of multi-cellularity, and the biogeography of unicellular diazotrophs and their role in biogeochemical cycles. Evaluating these broader implications will require a more complete understanding of the mechanisms and economics of material transfers within colonies, and the environmental factors that influence and sustain them.

Our results suggest that the exchange of newly fixed N within colonies is key to reducing population carbon costs, potentially explaining why the cells are often observed in aggregations (Fig. 1). However, the mechanisms of ${{{\mathrm{NH}}}}_4^ +$ transfer between cells and its overall efficiency (*E*_*N*_) within each colony remain poorly constrained. It is likely that the surface:volume ratio of the cell and the size of aggregated colonies can both influence *E*_*N*_. The diffusivity between the cellular spaces, might be affected by the production of extracellular polymeric substances. Also, the uptake properties of the cells (i.e. the maximum uptake rate and the half saturation constants of ${{{\mathrm{NH}}}}_4^ +$) influence how effectively they obtain ${{{\mathrm{NH}}}}_4^ +$. For example, if the N is transported with intercellular transporters, *E*_*N*_ would decrease considerably. To understand what regulates population heterogeneity of N_2_ fixation, it may be useful to examine the heterogeneity of N_2_ fixation under varying growth conditions, including different ambient ${{{\mathrm{NH}}}}_4^ +$ and O_2_ concentrations. Also, it is possible that N_2_ fixation is tied to specific phase of the cell cycle, which requires further experiments.

Recent ocean ecological and biogeochemical models simulate various functional groups of diazotrophs including unicellular types^55,56^, but diazotrophs within the same functional groups are generally represented as a uniform metabolic population. Given the observed bimodality of N_2_ fixation and its impact on C cost, our study suggests that resolving such heterogeneity and its underlying causes may be essential to simulating the ocean ecosystems and predicting the niche of unicellular diazotrophs. In particular, the dependence of C savings on cellular growth rate would help to test the model predictions for expanded vertical habitat. On the other hand, our model shows that population C savings are relatively insensitive to uncertainties in *E*_*N*_, especially at low growth rate, where the rate of N assimilation becomes small and costs of respiratory protection dominate (Fig. 7c and Supplementary Fig. 5). Thus, while the expansion of vertical niche depends on the growth rate dependence of C savings, it appears robust to uncertainty in N transfer efficiency, *E*_*N*_.

Given the ubiquity of phenotypic heterogeneity^57^ and inter-cellular cooperation^58^, metabolic heterogeneity may be a general strategy for maximizing fitness among diazotrophic cyanobacteria. It remains an open question whether filamentous diazotroph *Trichodesmium* separates N_2_ fixing cells (diazocytes) and cells responsible for photosynthesis^8,59–61^ or not^35,62^. If *Trichodesmium* separates N_2_ fixation and photosynthesis on cellular level, the observation of heterogeneity of N_2_ fixation in both *Crocosphaera* and *Cyanothece* together with the heterogeneity in N_2_ fixation in *Trichodesmium*^35^ suggest an evolutionary relationship between unicellular and filamentous diazotrophs. However, it remains an open question whether there is connection between heterogeneity in N_2_ fixation in unicellular diazotrophs and multicellular diazotrophs, as well as whether unicellular or multicellular diazotrophs evolved first in the cyanobacterial lineage^63–65^. The finding that heterogeneity in N_2_ fixation occurs in both unicellular and multicellular diazotrophs may support the hypothesis that the division of labor is a key factor driving multi cellular cooperation in evolutionary transitions^66,67^.

## Methods

### Phytoplankton cultures

A *Crocosphaera* strain isolated from the surface of the western subtropical Pacific^68^ was grown in a continuous 1.2 L culture in N-free medium. To closely represent their habitat (the euphotic zones of subtropical gyres), the culture was maintained in a chemostat with a dilution rate 0.20 d^−1^ (40% of the maximum growth rate), at a temperature of 26 °C, an irradiance of 200 µmol photons m^−2^ s^−1^, and a dark:light cycle of 12:12 h (1D to 12D, 1 L to 12 L). The beginning of the dark period was considered time 0 (0D). The N-free medium was prepared from seawater collected from the surface of the western North Pacific Ocean (34°20’N, 138°40’E), enriched with 20 µM of NaH_2_PO_4_, f/2 vitamins, and f/2 trace metals^69,70^. *Cyanothece* sp. ATCC51142 was grown in a 1.0 L culture in N-free ASP2 medium^71^ which contains 28.7 µM of K_2_HPO_4_, a temperature 26 °C, an irradiance of 400 µmol photons m^−2^ s^−1^, and a dark:light cycle of 12:12 h (1D to 12D, 1 L to 12 L) at growth rate (*μ*) of 0.30 d^−1^.

### ^15^N and ^13^C uptake

N_2_ fixation was measured following the method described by Mohr et al.^23^. Briefly, N-free medium was degassed and rapidly transferred to 125 mL glass bottles with minimal agitation until the maximum volume of the bottles was reached. These were septum-capped and enriched by injecting 1 mL of ^15^N_2_ gas (99.8 atom% ^15^N, lot #11059; SI Science Co., Ltd., Tokyo, Japan) into the 24 vials. Previous study confirmed no contamination of ^15^${{{\mathrm{NO}}}}_3^ -$ and ^15^${{{\mathrm{NH}}}}_4^ +$ in the ^15^N_2_ gas^72^. To observe ^15^N and ^13^C uptake, 0.5 mL of the ^15^N_2_-enriched medium was then added to 9.0 mL of *Crocosphaera* cultures (4.1 × 10^5^ cells mL^−1^) harvested from the continuous culture in 10 mL serum vials, to a final N_2_ enrichment of 5.5 atom% and 0.5 mL NaH^13^CO_3_ was injected simultaneously to a final enrichment of 9.7 atom%. These vials (*n* = 24) were sealed with crimp-seal butyl tube closures to eliminate headspace and air bubbles, preventing dilution of ^15^N_2_ with atmospheric ^14^N_2_. The vials were incubated under the same conditions as previously described and harvested one vial every hour beginning at the start of the dark period (6 PM), and split into three aliquots for NanoSIMS, PON and mass spectrometry, and flow cytometry. Samples prior to isotope injection were also collected and analyzed as time 0. Samples at 4 L were lost. Cells observed under NanoSIMS analysis were from 7 to 37 cells at each time point.

In *Cyanothece*, ^15^N and ^13^C uptake were analyzed as described for *Crocosphaera*, except for small differences in the source of ^15^N_2_ gas (98 atom% ^15^N, lot# MBBB0968V; Sigma-Aldrich, St. Louis, Missouri, USA), culture volume (4.0 mL of 1.7 × 10^6^ cells mL^−1^ in 5 mL serum vials), final enrichment (13.6 atom% and 1.7 atom% for ^15^N and ^13^C, respectively), sampling frequency (2 h), and the 87 to 220 cells were analyzed per each time point. Contamination of dissolved inorganic nitrogen in N_2_ was not analyzed in the ^15^N gas.

### NanoSIMS imaging

Cells (1 mL) were fixed in 2.0% w/v glutaraldehyde, and collected using 0.2-µm Isopore^TM^ GTTP Millipore Membrane filters (Merck Millipore, Billerica, Massachusetts, USA), which were then washed with Milli-Q ultrapure water and stored at −20 °C until further processing. For analysis, samples were sputtered with gold and secondary ions were imaged in 5 or 10 serial images (layers) on a NanoSIMS 50 (Cameca, Gennevilliers, France) to quantify ^12^C, ^13^C, ^12^C^14^N, and ^12^C^15^N in 7 to 220 cells per time point, following earlier studies^34,73^. Secondary ions were generated by pre-sputtering with a 300 or 500 pA Cs^+^ beam before scanning a raster of 256 × 256 pixels (10–15 µm^2^ total raster size) with a 1.7–1.8 pA Cs^+^ primary beam. Ratios of ^15^N:^14^C (inferred from the ^12^C^15^N/^12^C^14^N) and ^13^C:^12^C (^13^C/^12^C) are shown in the results (Figs. 1–4, Supplementary Fig. 1 and Supplementary Table 1). The system was tuned for ~9,000 mass resolving power to overcome isobaric interference, and confirmed against isotopic ratios obtained in organic particulates determined by Flash EA elemental analyzer (Thermo Electron Corporation, Waltham, Massachusetts, USA) coupled to a DELTA^plus^ XP mass spectrometer (Thermo Electron Corporation, Waltham, Massachusetts, USA) (Supplementary Fig. 1).

### Elemental analysis and mass spectrometry

Cells (8 mL) were collected on Whatman GF/F filters (GE Healthcare UK Ltd., Little Chalfont, Buckinghamshire, United Kingdom) pre-combusted at 450 °C for 6 h, and frozen at −20 °C until further processing. For analysis, filters were dried at 50 °C overnight, exposed to HCl fumes for 2 h, and then dried again. The concentration and isotopic composition of total particulate organic C and N were measured on a Flash EA elemental analyzer (Thermo Electron Corporation, Waltham, Massachusetts, USA) coupled to a DELTA^plus^ XP mass spectrometer (Thermo Electron Corporation, Waltham, Massachusetts, USA). The abundance of ^13^C and ^15^N were expressed as δ^13^C (or δ^15^N) (‰): δ^13^C (δ^15^N) = [(*R*_sample_/*R*_standard_) −1] * 1000. Lower limit of the detection of the Flash EA elemental analyzer (Thermo Electron Corporation, Waltham, Massachusetts, USA) is 0.005 mg N (Supplementary Table 3).

### Flow cytometry

Samples (4.5 mL) were fixed with 0.5% w/v glutaraldehyde, and stored at −80 °C until being counted on a PASIII flow cytometer (Partec GmbH, Münster, Germany) equipped with 10 mW argon ion lasers.

### Calculation of carbon and nitrogen uptake rates

Images obtained by NanoSIMS were processed in ImageJ^74^ following methods described by Popa et al.^34^. Briefly, the mean isotopic compositions in each cell, delineated by the ^12^C^14^N images, were integrated over 5 or 10 serial images, corrected against reference standards, and converted to percentage uptake with a measurement precision of 0.8–1.5%. Cells with a ^12^C^15^N:^12^C^14^N (^15^N:^14^N) ratio exceeding 2 standard deviations above the average at time 0 (at which ^15^N:^14^N was 3.8 ‰ for *Crocosphaera*, 4.0 ‰ for *Cyanothece*) were considered ^15^N-enriched. Similarly, cells with a ^13^C:^12^C ratio exceeding 2 standard deviations above the mean at time 0 (at which ^13^C:^12^C was 9.8 ‰ for *Crocosphaera*, 11.8 ‰ for *Cyanothece*) were considered ^13^C-enriched.

The rate of N_2_ fixation was defined as the change in % ^15^N h^−1^ relative to the initial measurement. Per-cell net N uptake rates (*ρ*; fmol N cell^−1^ h^−1^) were calculated using a method adapted from Popa et al.^34^, described in [Eq. 2].

2 $$\rho = Fx_{net} \times CellQ/{{\Delta }}t$$

where *Fx*_*net*_ is the ratio between ^15^N in a cell after*Δt* and the initial ^15^N content, and *CellQ* is the cellular N quota calculated as the sum of particulate organic ^15^N and ^14^N normalized to the cell density. As N_2_ fixation in *Crocosphaera* occurs only at night^21,29^, ^15^N enrichment in the dark (0–12 h) and during light (13–24 h) were treated as N_2_ fixation and re-uptake of excreted dissolved ^15^N, respectively.

### Statistics and reproducibility

^15^N:^14^N ratios were compared by one-way ANOVA^39^ with 25 time points as factor levels, and individual cells in a sample as independent replicates. Differences were considered significant if *p* < 0.05. Heterogeneity was defined by the coefficient of variation (CV; ref. ^41^):

3 $$CV = 100 \times \sigma / {\bar{x}}$$

where ${\bar{x}}$ is the mean and *σ* is the standard deviation among the cells. Normality assumptions were confirmed after logarithmic transformation (*p* > 0.05 by K-S test, *n* = 7–37 for *Crocosphaera*, *n* = 87–220 for *Cyanothece*) and residuals had a mean of zero. Dunnett’s T-3 multiple comparisons^75^ were used to compare background ratios.

To compute the bimodal separation, we first fit the sum of two Gaussian distributions to the histogram^42^:

4 $$F_B\left( x \right) = A_1exp\left\{ { - \left( {x - \\ {\bar{x}} _1} \right)^2/2\sigma _1^2} \right\} + A_2exp\left\{ { - (x - \\ {\bar{x}} _2)^2/2\sigma _2^2} \right\}$$

where *F*_*B*_(*x*) is frequency of *x*, *A*_*i*_ is amplitude, ${\bar{x}}$_*i*_ is mean and *σ*_*i*_ is standard deviation (*i* = 1 or 2 and ${\bar{x}}$_2_ > ${\bar{x}}$_1_). We obtain *A*_*i*_, ${\bar{x}}$_*i*_ and *σ*_*i*_ with Metropolis Algorithm^76,77^, that minimizes the sum of square error between [Eq. 4] and the histogram. Based on values of ${\bar{x}}$_*i*_ and *σ*_*i*_, obtained, we calculate the bimodal separation:

5 $$S = \frac{{\\ \bar{x} _2 - \\ \bar{x} _1}}{{2\sigma _1 + 2\sigma _2}}$$

To examine the statistical significance of the difference between N and C uptake, we use the curve fitted to ^15^N:^14^N, and re-fitted to ^13^C:^12^C, by maintaining the original relative relationship between *A*_1_ and *A*_2_, ${\bar{x}}$_1_ and ${\bar{x}}$_2_, and *σ*_1_ and *σ*_2_ and value of *S* obtained based on ^15^N:^14^N of the same diazotroph. The *p* value is obtained based on the difference between the data of ^13^C:^12^C and the fitted curve as a null hypothesis.

To compute the deviation from the normal distribution, we applied the following procedure. If variation in the rate of C or N uptake is randomly distributed among cells of a population with a constant mean rate, we expect the probability density of C and N uptake follows the normal distribution^78^:

6 $$E(x) = \frac{A}{{\sqrt {2\pi \sigma ^2} }}exp\left\{ { - \frac{{\left( {x - \\ \bar{x} } \right)^2}}{{2\sigma ^2}}} \right\}$$

where *E*(*x*) is the expected probability density for value *x* based on the normal distribution, *A* is the total area of the histogram, *σ* is the observed standard deviation, and ${\bar{x}}$ is the observed mean value. If the C or N uptake of the population is heterogeneous, we expect stronger deviation from [Eq. 6]; we calculate the deviation from the Chi squared (*χ*^2^) statistic^79^, normalized by the sample number:

7 $$Dev = \frac{{\chi ^2}}{n} = \frac{1}{n}{\sum} {\frac{{\left( {O(x) - E(x)} \right)^2}}{{E(x)}}}$$

where *O*(*x*) is observed probability density for the value *x*. The normalization by *n* makes results with different sample numbers comparable (here *Crocosphaera* and *Cyanothece*).

Reproducibility was confirmed by analyzing 7 to 37 independent *Crocosphaera* cells, and 87 to 220 independent *Cyanothece* cells (Supplementary Table 1).

### Numerical model of heterogeneous metabolisms

To represent heterogeneous metabolisms within a single clonal population of unicellular diazotrophs, we have modified the Cell Flux Model of diazotrophs^44^ by simulating two types of cells; N_2_-fixing and non-N_2_ fixing (Fig. 5). The model resolves a coarse-grained metabolic flux network based on mass, electron and energy (ATP) balance. These balances quantify stored C use for 3 cellular functions: biosynthesis, electron donation for N_2_ fixation, and respiration. Respiration can be further classified into three uses; respiration for biosynthesis, for N_2_ fixation and for respiratory protection (Fig. 5). The model was parameterized for *Crocosphaera* based on a respiration budget^43^ by reducing the diffusivity of cell membranes^44^. We use cellular N of 30 fmol N cell^−1^ and a diameter of 3μm and temperature of 28 °C to better represent *Crocosphaera* (strain WH8501) in Großkopf and Laroche^43^, which gives the diffusivity coefficient of the membrane of 1.51  × 10^−5^, slightly higher than previously estimated (1.38 × 10^−5^). To represent *Crocosphaera* in this study (strain PS0609A) we used a cell diameter of 5 µm (based on Fig. 1 and Sohm et al.^80^ for a larger size class), a cellular N of 60 fmol cell^−1^, and the maximum N_2_ fixation rate of 6.1 fmol cell^−1^ h^−1^. To represent the laboratory condition, we applied temperature *T* = 26 °C and assume saturated O_2_ concentration [O_2_] = 208 µM (ref. ^81^), and *μ* = 0.20 d^−1^ (when *µ* is constant). We have used a uniform growth rate among cells following previous studies^82–86^.

### Application of the model to one dimensional water column

To simulate the light attenuation in the one-dimensional water column, we used Beer’s law:

8 $$I\left( z \right) = I_0e^{ - kz}$$

where *I*(*z*) is the light intensity (µmol m^−2^ s^−1^) at the depth of *z* (m), *I*_0_ is the light intensity at the surface (µmol m^−2^ s^−1^), and *k* is the extinction coefficient (m^−1^). To simulate the photosynthesis rate by *Crocosphaera*, we adapt a commonly used equation with saturating light based on Target theory^85,87^:

9 $$P(I) = P_{{{\mathrm{max}}}}(1 - e^{ - I/I_0^P})$$

where *P*(*I*) is the rate of photosynthesis (fmol C cell^−1^ h^−1^) at the light intensity of *I*, *P*_max_ is the maximum photosynthesis rate (fmol C cell^−1^ h^−1^), $I_0^P$ is the reference light intensity at which *P* becomes (*e* − 1)/*e*. Then, with the Cell Flux Model, we find the growth rate *µ* (d^−1^) where *C*_*S*_(*µ*) = *P*(*I*), where we use *E*_*N*_ = 0.2 and *f*_*N*_ = 0.5 for the population with heterogeneous N_2_ fixation and *f*_*N*_ = 1 for the population with homogeneous N_2_ fixation. The loss of C to the environment is assumed equal for both of these populations. We consider a simple 12:12 (h) light:dark cycle, at which photosynthesis occurs only during the light period and N_2_ fixation and respiratory protection occur only during the dark periods. We apply *I*_*0*_ = 1000 and *k* = 30^−1^ to resemble observed depth profile of light in the subtropical gyres^50,88^, and *P*_*max*_ = 7 and $I_0^P = 100$ where the simulated maximum growth rate becomes close to the highest side of the observed range^48^ and MVD of the population of heterogeneous N_2_ fixation becomes close to 125 (m), below which the *nifH* copies of *Crocosphaera* is observed to drop considerably.

### Reporting summary

Further information on research design is available in the Nature Research Reporting Summary linked to this article.

## Supplementary information

Supplemental Information

Supplementary Data

Description of Additional Supplementary Files

Reporting Summary

## Data Availability

The data used to generate the graphs presented in the main figures can be found as Supplementary Data 1. All other data that support the findings of this study are available on request from the corresponding author (TM).

## Code availability

The model has been written in Python 3 and freely available in Zenodo at https://zenodo.org/record/1251956 (10.5281/zenodo.1251956).
