# Author Correction: Heterogeneous nitrogen fixation rates confer energetic advantage and expanded ecological niche of unicellular diazotroph populations

**DOI:** 10.1038/s42003-023-05007-6

**Published:** 2023-06-07

**Authors:** Takako Masuda, Keisuke Inomura, Naoto Takahata, Takuhei Shiozaki, Yuji Sano, Curtis Deutsch, Ondřej Prášil, Ken Furuya

**Affiliations:** 1 Department of Aquatic Bioscience, The University of Tokyo, Yayoi, Bunkyo, Tokyo 113-8657 Japan; 2 Institute of Microbiology, The Czech Academy of Sciences, Opatovický mlýn, 379 01 Třeboň, Czech Republic; 3 School of Oceanography, University of Washington, Seattle, WA USA; 4 Atmosphere and Ocean Research Institute, The University of Tokyo, Kashiwanoha, Kashiwa-shi, Chiba, 277-8564 Japan; 5 Present Address: Global Change Research Institute, The Czech Academy of Sciences, 664 24 Drásov, Olomouc, Czech Republic; 6 Present Address: Graduate School of Science and Engineering, Soka University, Tangi, Hachioji, Tokyo 192-8577 Japan

**Keywords:** Microbiology, Microbial ecology, Cellular microbiology

Correction to: *Communications Biology* 10.1038/s42003-020-0894-4, published online 14 April 2020.

In the original version of the Article, in the last paragraph of the Methods section, one of the parameters and its value was incorrectly described as “*z*_0_ = 30” when it should have read “*k* = 30^−1^”.

This has now been corrected.

