# Supplementary material for: Heterogeneous nitrogen fixation rates confer energetic advantage and expanded ecological niche of unicellular diazotroph populations
Source: Commun Biol. 2020 Apr 14;3:172. doi: 10.1038/s42003-020-0894-4 (PMC7156374; doi:10.1038/s42003-020-0894-4)
Supplement: Supplementary file 1 — Supplemental Information [file 42003_2020_894_MOESM1_ESM.pdf]

Supplementary Figures

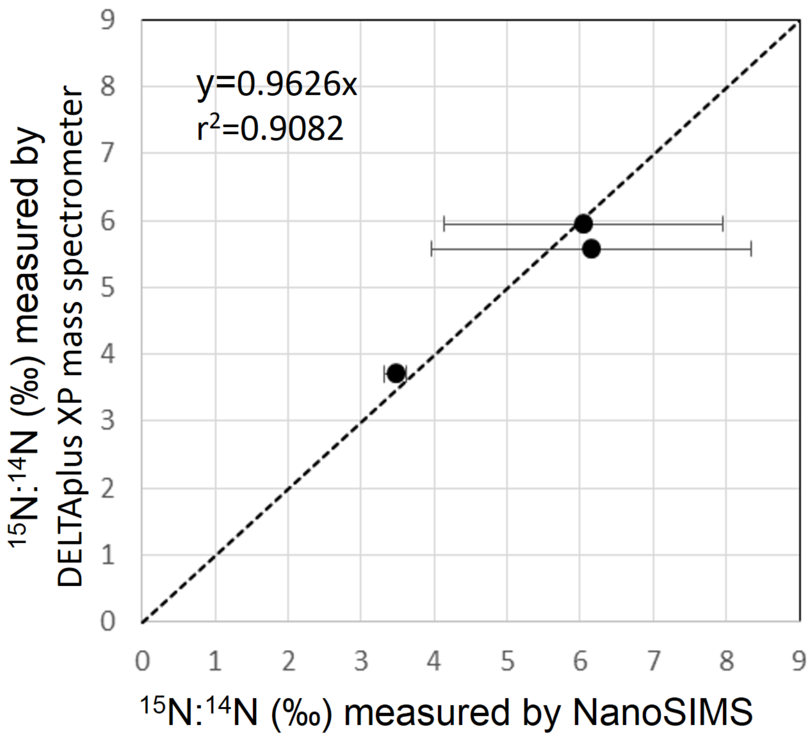

Supplementary Figure 1. Correlation between average ^{15}N:^{14}N ratio with SD measured by NanoSIMS and which measured by a DELTA^{plus} PX mass spectrometer. Dashed line represent $y = x$.

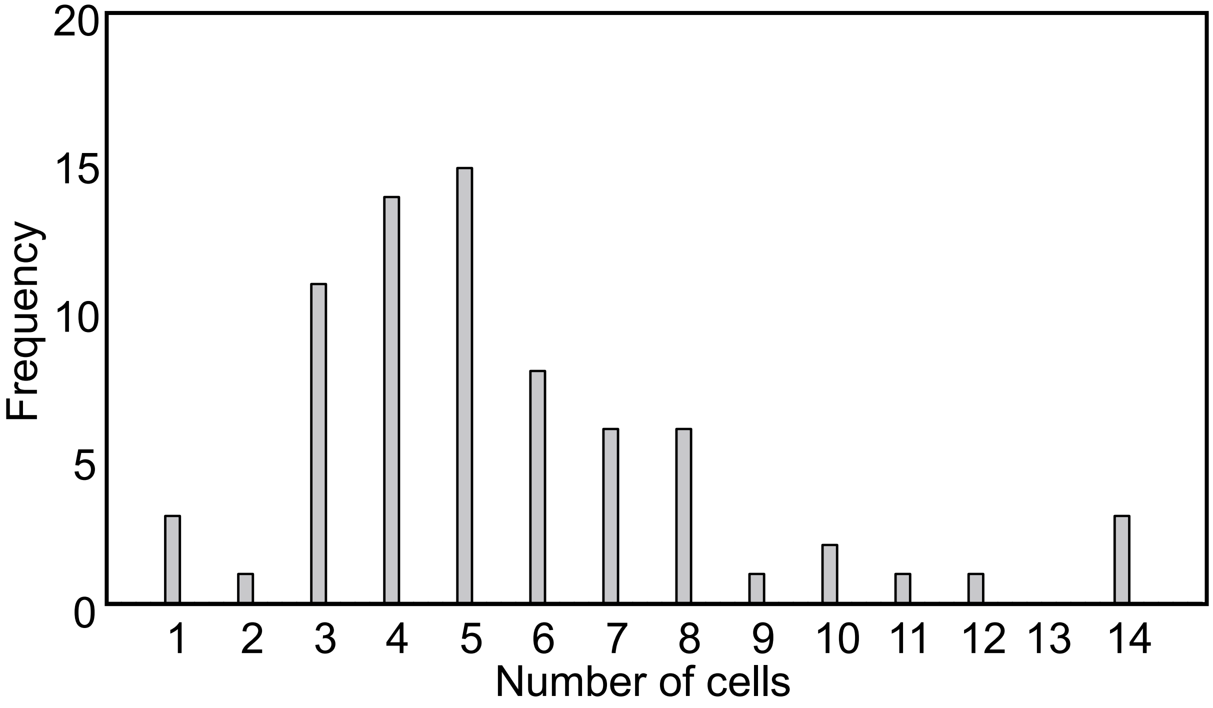

Supplementary Figure 2. Frequency distribution of the number of cells in *Crocosphaera* aggregation, evaluated from NanoSIMS images. In total 70 cells were observed.

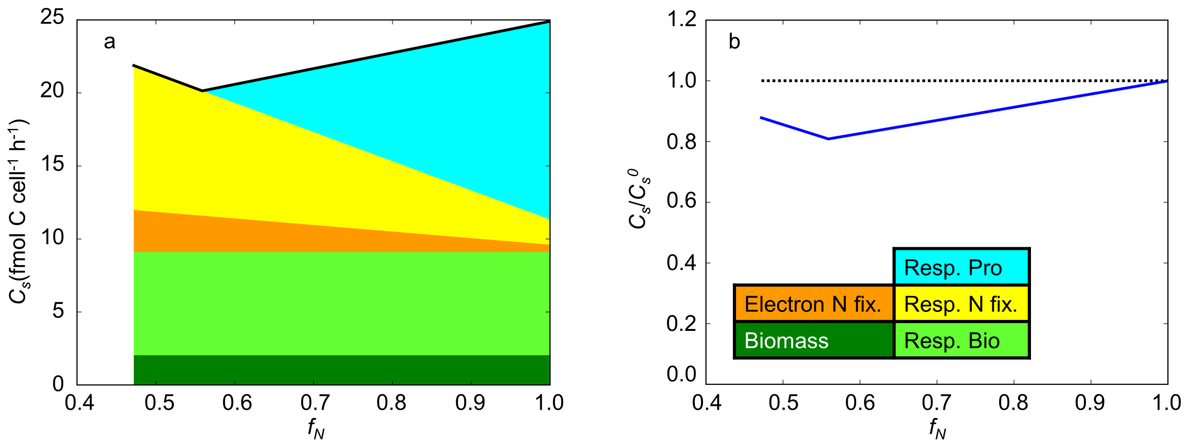

Supplementary Figure 3. Carbon use of heterogeneous population, C_S and C_S relative to non-heterogeneous population C_S^0 for $E_N = 0.1$. (a) C_S for various f_N. (b) C_S/C_S^0 for various f_N. For (a) the legend in (b) shows the colors used for each fluxes; Dark green, biosynthesis; Bright green, respiratory energy production for biosynthesis; Orange, electron donation for N_2 fixation; Yellow, respiratory energy production for N_2 fixation; Cyan, respiratory protection. See Fig. 5 for more detail where similar colors are used for each C flux. Black solid line at the top of (a) represents the total C flux. Black dotted line in (b) is for $C_S/C_S^0 = 1$. $f_N = 0.5$, $E_N = 0.1$, and $\mu = 0.2$ (d^{-1}) unless they are variable on the x axes. Temperature T = 26˚C and O_2 concentration in the environment $[O_2] = 208$ μM, representing saturated concentration at this temperature and salinity of 35ppt[1]

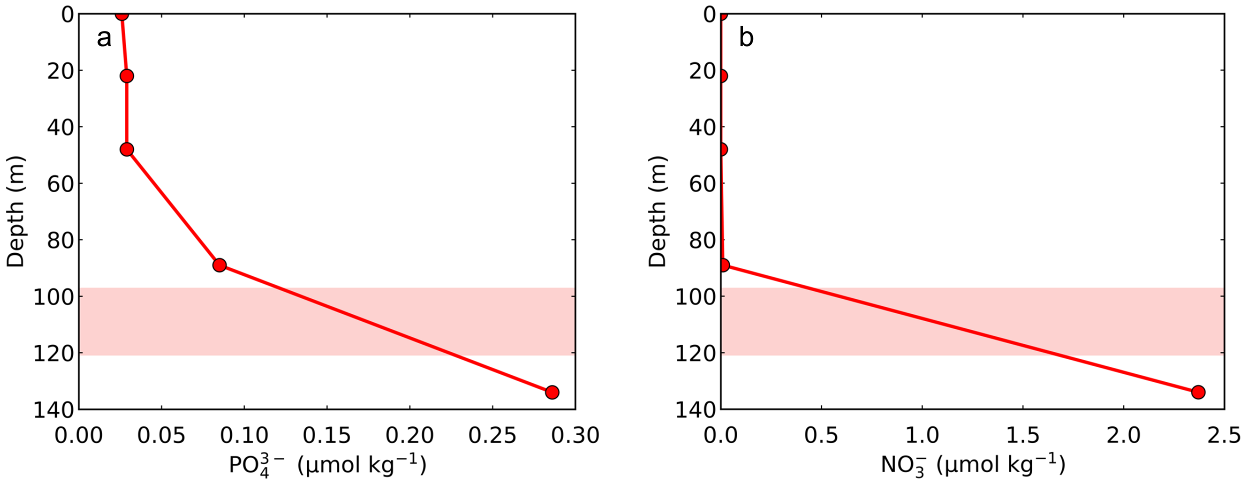

Supplementary Figure 4. Depth profile of (a) PO_4^{3-} and (b) NO_3^- concentrations in the South Pacific at 25 º S 170 º W, where highest abundance *nifH* gene of *Crocosphaera* is observed during the cruise[2]. The red shading is the model predicted difference of MVD (the depth where μ becomes zero) between the two different populations in (see Fig. 8a).

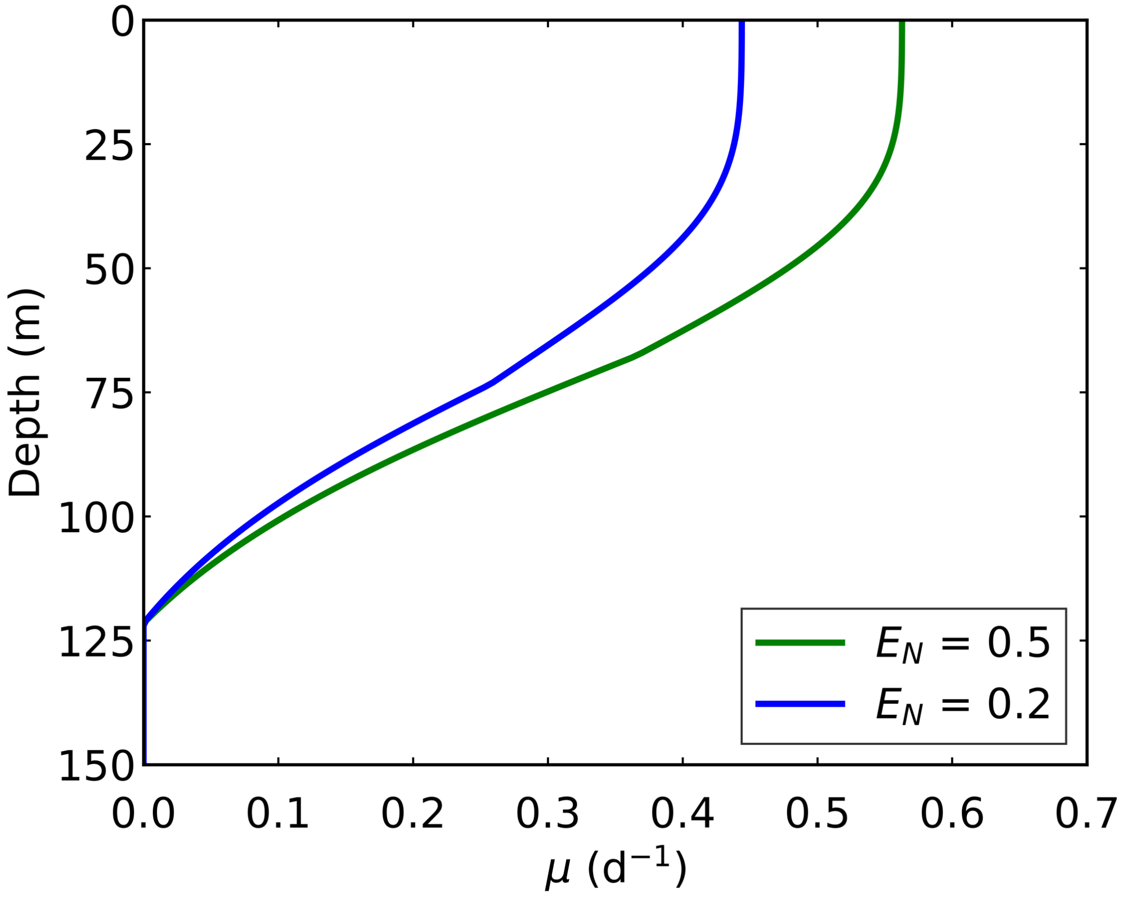

Supplementary Figure 5. Influence of E_N on the prediction of light limited growth rate (μ) of *Crocosphaera* for various depths.

Supplementary Tables

Supplementary Table 1. Temporal change of observed number of cells, average (AV), standard deviation (SD), coefficient of variation (CV) of (a)^{13}C:^{12}C, ^{15}N:^{14}N, and (b) ^{13}C fixation rate and ^{15}N fixation rate for *Crocosphaera* and *Cyanothece*.

a	*Crocosphaera*							*Cyanothece*						
		^{13}C:^{12}C			^{15}N:^{14}N				^{13}C:^{12}C			^{15}N:^{14}N		
Time	Number of cell	AV	SD (‰)	CV (%)	AV	SD (‰)	CV (%)	Number of cell	AV	SD (‰)	CV (%)	AV	SD (‰)	CV (%)
0	22	8.8	0.5	5.6	3.5	0.1	4.2	105	11.0	0.4	3.4	3.6	0.2	4.8
1	14	8.9	0.4	4.3	3.4	0.1	3.1	0						
2	15	8.6	0.4	5.2	3.4	0.1	4.0	141	11.2	0.3	2.9	4.8	2.1	44.2
3	15	9.0	0.4	4.0	3.5	0.1	2.8	0						
4	15	9.3	0.3	3.7	3.8	0.3	8.7	158	11.0	0.6	5.5	6.3	3.5	56.1
5	11	9.2	0.3	3.7	3.8	0.4	9.2	0						
6	16	9.0	0.5	5.2	4.4	1.0	23.6	87	11.9	0.5	4.4	8.3	4.4	53.8
7	10	9.1	0.2	2.7	5.1	1.3	24.9	0						
8	16	8.9	0.3	3.0	4.3	1.2	28.2	146	11.7	0.5	4.4	9.0	4.5	50.5
9	22	9.3	0.2	2.3	4.9	1.6	32.7	0						
10	13	8.9	0.3	3.0	4.5	1.3	29.2	141	12.0	0.6	5.2	9.9	4.6	46.4
11	12	8.8	0.2	2.3	5.9	1.8	29.7	0						
12	18	9.2	0.3	2.9	6.0	1.9	31.4	103	12.2	0.6	4.7	8.6	4.8	56.2
13	9	8.9	0.2	1.8	6.6	2.3	34.6	0						
14	20	8.7	0.3	3.0	5.0	1.8	35.8	173	18.6	2.6	14.2	9.9	4.5	45.7
15	33	8.7	0.5	6.2	4.3	1.3	30.6	0						
16	0							176	11.8	0.7	5.6	9.0	5.0	55.3
17	37	9.1	0.4	4.7	5.2	1.6	31.0	0						
18	16	9.3	0.5	5.7	5.0	2.0	39.7	169	38.6	9.3	24.0	10.1	4.6	46.0
19	16	8.9	0.4	5.0	5.2	2.5	48.0	0						
20	10	10.4	1.0	9.8	5.5	1.4	25.4	220	35.1	14.1	40.1	9.3	4.6	48.9
21	12	10.8	0.5	4.8	5.6	1.9	34.9	0						
22	10	10.4	0.7	6.8	6.1	1.7	28.6	165	42.6	10.9	25.6	10.5	4.8	45.5
23	7	11.3	1.0	8.8	6.2	2.2	36.3	0						
24	26	10.1	1.1	10.6	4.5	1.7	37.9	152	47.6	12.2	25.6	10.8	5.2	48.4

b	*Crocosphaera*							*Cyanothece*						
		^{13}C fixation rate			$^{15}N_2$ fixation rate				^{13}C fixation rate			$^{15}N_2$ fixation rate		
Time	Number of cell (cells)	AV (fmol C cell^{-1}h^{-1})	SD	CV (%)	AV (fmol N cell^{-1}h^{-1})	SD	CV (%)	Number of cell (cells)	AV (fmol C cell^{-1}h^{-1})	SD	CV (%)	AV (fmol N cell^{-1}h^{-1})	SD	CV (%)
0	22							105						
1	14	10.47	102.22	976	-2.08	7.83	376							
2	15	-32.43	56.66	175	-3.14	5.01	159	141	54.87	8.20	15	4.18	7.81	187
3	15	12.70	32.14	253	0.10	2.40	2477							
4	15	29.36	23.33	79	6.93	6.21	90	158	25.77	7.82	30	4.97	6.60	133
5	11	19.65	17.97	91	5.15	5.19	101							
6	16	5.67	20.54	362	11.77	12.90	110	87	24.26	4.42	18	5.75	5.54	96
7	10	8.58	9.27	108	17.29	13.45	78							
8	16	2.67	8.75	327	7.23	11.11	154	146	17.26	3.32	19	4.97	4.24	51
9	22	13.05	6.39	49	11.68	13.17	113							
10	13	2.26	7.00	310	7.80	9.80	126	141	15.40	3.22	21	4.64	3.43	46
11	12	-1.93	4.85	251	16.53	11.86	72							
12	18	7.27	5.88	81	15.93	11.77	74	103	13.77	2.48	18	3.10	3.02	56
13	9	1.50	3.20	213	18.03	13.09	73							
14	20	-2.23	4.99	224	7.96	9.44	119	173	35.34	9.85	28	3.34	2.42	46
15	33	-2.97	9.50	320	4.20	6.54	156							
16	0							176	8.95	2.12	24	2.51	2.33	55
17	37	4.86	6.72	138	7.47	7.01	94							
18	16	6.23	7.76	125	6.40	8.21	128	169	86.72	27.71	32	2.68	1.93	46
19	16	0.70	6.20	890	6.62	9.67	146							
20	10	21.22	13.60	64	7.56	5.18	68	220	68.93	37.46	54	2.12	1.71	49
21	12	24.94	6.56	26	7.42	6.87	93							
22	10	18.82	8.57	46	8.71	5.85	67	165	76.95	25.27	33	2.33	1.63	46
23	7	27.53	12.22	44	8.63	7.06	82							
24	26	14.22	11.80	83	3.09	5.23	170	152	85.35	26.98	32	2.24	1.64	48

Supplementary Table 2. Maximum cellular N_2 fixation for *Crocosphaera watsonii*. Note different unit of N_2 fixation rate. When exact value of N_2 fixationrate is not described in the text, N_2 fixation rate is described with "~", which is read from graph. In the method column, AR shows acetylene reduction method, ^{15}N shows isotope labeling of $^{15}N_2$, MS shows mass spectrometry.

strain	Light intensity (μmol photons $s^{-1}m^{-2}$)	LD cycle	Temp. (°C)	Culturing method	Medium	Maximum cellular N_2 fixation rate	Method	
WH8501	50	14L10D	25	Batch	SNAX	~0.17 (fmol N $cell^{-1}$ h^{-1})	AR	Webb et al., 2009
WH0003	50	14L10D	25	Batch	PMP	~0.57 (fmol N $cell^{-1}$ h^{-1})	AR	Webb et al., 2009
WH8501	50	12L12D	28	Batch	YBCII	~1.6 (fmol C_2H_2 $cell^{-1}$ h^{-1})	AR	Mohr et al., 2010
WH8501	50	12L12D	26	Batch	YBCII	~5 (fmol N_2 $cell^{-1}$ h^{-1})	AR	Shi et al., 2010
WH8501	180	12L12D	27	Batch	YBCII	1.05 (fmol N_2 $cell^{-1}$ h^{-1})	AR	Dekaezemacker and Bonnet, 2011
WH8501	?	14L10D	?	?	SO	~17 (fmol C_2H_2 $cell^{-1}$ h^{-1})	AR	Saito et al., 2011
WH8501	130	12L12D	27	Chemostat D=0.2d^{-1}	YBCII	11.5 (fmol N $cell^{-1}$ h^{-1})	AR	Dron et al., 2011
WH8501	130	12L12D	27	Chemostat D=0.2d^{-1}	YBCII	23.3 (fmol N $cell^{-1}$ h^{-1})	AR	Dron et al., 2011
WH8501	150	12L12D	28	Batch	YBCII	2.14 ± 0.34 (fmol C_2H_2 $cell^{-1}$ h^{-1})	AR	Großkopf and La Roche, 2012
WH8501	80	12L12D	27	Batch	YBCII	~0.15 (fmol C_2H_2 $cell^{-1}$ h^{-1})	AR	Knapp et al., 2012
WH0003	18-300	12L12D	28	Batch	Aquil	22 (fmol N $cell^{-1}$ h^{-1})	AR	Garcia et al., 2013
PS0609A	200	12L12D	26	Chemostat D=0.20d^{-1}	Modified f/2	7.3 ± 0.7 (fmol C_2H_2 $cell^{-1}$ d^{-1})	AR	Masuda et al., 2013
PS0609A	200	12L12D	26	Chemostat D=0.20d^{-1}	Modified f/2	7.4 ± 0.6 (fmol C_2H_2 $cell^{-1}$ d^{-1})	$^{15}N_2$-MS	Masuda et al., 2013
WH8501	70-100	12L12D	28	Batch	YBCII	~1.3 (fmol N $cell^{-1}$ h^{-1})	AR	Mohr et al., 2013
WH8501	70-100	12L12D	28	Batch	YBCII	~1.1 (fmol N $cell^{-1}$ h^{-1})	$^{15}N^{2}$-NanoSIMS	Mohr et al., 2013
WH8501	150	12L12D	27.5	Batch	YBCII	7.8 ± 2.4 (fmol N $cell^{-1}$ h^{-1})	$^{15}N_2$-MS	Jacq et al., 2014
WH0003	175	12L12D	28	Batch	YBCII	135.4 ± 1.2 (fmol N $cell^{-1}$ h^{-1})	AR	Garcia and Hutchins, 2014
PS0609A	200	12L12D	26	Chemostat D=0.2d^{-1}	Modified f/2	17.29 (fmol N $cell^{-1}$ h^{-1})	$^{15}N^{2}$-NanoSIMS	This study

Supplementary Table 3. The standard deviations (SD) of the $\delta^{15}N$ value measured N standard (IAEA-N-1, Ammonium Sulfate), whose range covered amount of N in samples.

AV (mg N)	SD (‰)
0.005	0.94
0.015	0.43
0.046	0.16
0.092	0.09
0.135	0.015

References

1 Benson, B. B. & Krause, D. The concentration and isotopic fraction of oxygen dissolved in freshwater and seawater in equilibrium with the atmosphere. *Limnol Oceanogr* **29**, 620-632 (1984)

2 Shiozaki, T. *et al.* Linkage between dinitrogen fixation and primary production in the oligotrophic South Pacific Ocean. *Global Biogeochemical Cycles* **32**, 1028-1044, doi:10.1029/2017gb005869 (2018)
