# Supplementary material for: Heterogeneous nitrogen fixation rates confer energetic advantage and expanded ecological niche of unicellular diazotroph populations
Source: Commun Biol. 2020 Apr 14;3:172. doi: 10.1038/s42003-020-0894-4 (PMC7156374; doi:10.1038/s42003-020-0894-4)
Supplement: Supplementary file 3 — Description of Additional Supplementary Files [file 42003_2020_894_MOESM3_ESM.pdf]

Description of Additional Supplementary Files

File Name: **Supplementary Data**

Description: Source data for the main figures (figures 2-4, 5- 8). The data for each figure is in a separate worksheet.
